# Experimental Evaluation of Carbonated Water Flooding Using Microfluidics and Coreflooding for Simultaneous EOR and CO_2_ Storage in Sandstone Reservoirs

**DOI:** 10.1021/acs.iecr.6c01713

**Published:** 2026-07-13

**Authors:** Sushobhan Pradhan, Khandaker Fahim Anjum, Prem Bikkina

**Affiliations:** School of Chemical Engineering, 7618 Oklahoma State University, Stillwater, Oklahoma 74078, United States

## Abstract

Carbon capture and storage (CCS) in depleted oil and gas reservoirs offers a sustainable pathway to mitigate greenhouse gas emissions through secure, long-term CO_2_ sequestration. Understanding CO_2_–brine–oil interactions at the pore and core scales is critical for predicting the fate of injected CO_2_ and optimizing storage efficiency. This study presents integrated microfluidic and coreflooding experiments to evaluate carbonated water/brine flooding (CWF) in both model and crude oil systems under varying pressure and salinity conditions. At low pressure (150 psig), CWF showed negligible change in residual oil saturation (*S*_or_) after waterflooding (WF). However, at elevated pressure (700 psig), *S*_or_ decreased by approximately 12–15%, demonstrating enhanced oil recovery (EOR) potential. CO_2_ flooding further reduced residual oil saturation at both low and high pressures. Interfacial tension (IFT) measurements revealed a linear decrease with increasing pressure and a linear increase with salinity for CO_2_-saturated brine–oil systems, whereas produced water–crude oil systems exhibited no significant pressure dependence. Coreflooding experiments confirmed that carbonated water injection enhances oil recovery with increasing pressure, and low-salinity systems outperform high-salinity counterparts. At low pressure (150 psig), increasing salinity reduced the total solubility trapping of CO_2_ in both brine and oil. In contrast, at high pressure (700 psig), increasing salinity enhanced total solubility trapping, a trend with significant implications for storage capacity estimates in hydrocarbon-bearing reservoirs. These findings elucidate the interdependence of CO_2_-EOR driven by oil swelling, viscosity reduction, and IFT reduction, and CO_2_ storage through solubility trapping, offering practical insights to optimize CCS and carbonated water injection strategies in hydrocarbon-bearing reservoirs.

## Graphical abstract

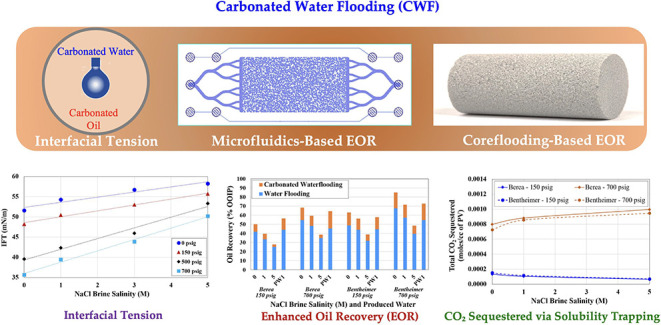

## 1 Introduction

The continued increase in atmospheric carbon dioxide (CO_2_) concentration due to anthropogenic activities has intensified global efforts to develop technologies capable of mitigating greenhouse gas emissions while maintaining reliable energy supply. Carbon capture, utilization, and storage (CCUS) has emerged as a central pillar of decarbonization strategies, particularly for energy-intensive sectors that are difficult to electrify or transition rapidly to alternative fuels. Geological storage of CO_2_ in deep subsurface formations, including saline aquifers and hydrocarbon reservoirs, offers a technically viable and scalable solution owing to the vast storage capacity of sedimentary basins and the presence of multiple long-term trapping mechanisms that promote secure containment of injected CO_2_.^1,2^ Within hydrocarbon reservoirs, where CO_2_ injection is often coupled with enhanced oil recovery (EOR), physicochemical interactions among CO_2_, formation brine, crude oil, and reservoir rock play a critical role in controlling multiphase flow behavior. In particular, interfacial tension (IFT) and wettability under reservoir conditions govern fluid distribution, capillary trapping, and displacement efficiency in porous media, thereby directly influencing both oil recovery and CO_2_ storage performance.^3^

Among geological storage options, hydrocarbon reservoirs are particularly attractive because of their proven sealing integrity, extensive subsurface characterization, and existing infrastructure. These factors significantly reduce technical uncertainty and capital expenditure associated with CO_2_ injection projects. When combined with EOR, CO_2_ injection can generate economic value while simultaneously storing substantial quantities of CO_2_ underground.^4^ This dual benefit has made CO_2_-EOR the most mature and widely deployed CCUS-related technology to date.

CO_2_-EOR improves oil recovery through a combination of physicochemical mechanisms, including oil swelling, viscosity reduction, IFT lowering, and, under favorable pressure–temperature conditions, near-miscible or miscible displacement.^5–7^ These mechanisms reduce capillary forces and enhance microscopic displacement efficiency, enabling the mobilization of residual oil that remains trapped after conventional waterflooding (WF). In parallel, the storage component of CCUS depends on the successful trapping of injected CO_2_ within porous subsurface formations. Multiple trapping mechanisms, including structural, residual, capillary, solubility (dissolution) in oil and brine, and, over longer time scales, mineral trapping, govern the long-term storage of CO_2_ in the reservoir.^8–10^ Although these mechanisms are coupled, their relative contributions evolve over different time scales depending on reservoir conditions such as pressure, temperature, fluid saturation, geochemical interactions, and structure of the pore network. Among these mechanisms, solubility trapping plays a dominant role during active CO_2_-EOR operations, particularly at elevated pressures where CO_2_ solubility in reservoir fluids is high.^11^

Despite its effectiveness, conventional gas-phase CO_2_ injection faces several operational challenges, including unfavorable mobility ratios, gravity override, and early gas breakthrough.^12–14^ These challenges reduce macroscopic sweep efficiency and motivate the development of alternative injection strategies such as water-alternating-gas (WAG), foam-assisted CO_2_ injection, and carbonated water flooding (CWF).^3,14,15^ Among these, CWF has gained increasing attention as a storage-oriented injection strategy because it avoids the introduction of a highly mobile free-gas phase and inherently enhances CO_2_ retention via solubility trapping.

Recent studies have further extended CWF beyond conventional continuous injection. Dang et al.^16^ introduced a soaking-enhanced CWF protocol in which a static soaking stage prolongs the interaction between carbonated water and reservoir fluids, allowing dissolved CO_2_ to diffuse more completely into the oil phase; this increased oil recovery from 56.4% to 73.4% and CO_2_ storage from 0.12 to 0.16 pore volumes at the highest CO_2_ concentration tested, with pressure equilibration during soaking redistributing CO_2_ into micropores and dead-end spaces. Similarly, Du et al.^17^ combined carbonated water with a surfactant to form active carbonated water and applied it in huff-n-puff mode in fractured low-permeability cores, achieving a recovery factor of 39.4% through ultralow IFT, strong wettability reversal toward water-wet conditions, and CO_2_ mass transfer-induced oil swelling. These studies underscore that CO_2_–oil contact time and CO_2_ partitioning into the oil phase are key levers governing both incremental recovery and CO_2_ retention during carbonated water-based injection.

At the core of CWF performance lies in the thermodynamics of CO_2_ solubility in hydrocarbons and aqueous phases. CO_2_ solubility governs the extent of oil swelling, viscosity reduction, and IFT alteration, as well as the amount of CO_2_ that can be immobilized through solubility trapping in residual reservoir fluids. Despite this central role, the coupled effects of CO_2_ solubility in reservoir fluids on oil recovery and carbon storage performance remain insufficiently understood, particularly under varying pressure and salinity conditions and across both pore- and core-scale systems. To address this gap, our recent studies systematically investigated CO_2_ solubility in brines and hydrocarbons relevant to CO_2_-EOR and storage applications. In the first study, the CO_2_ solubility was quantified in representative hydrocarbons over a wide pressure range relevant to subsurface injection. This work demonstrated strong density, pressure, and temperature-dependent CO_2_ dissolution in hydrocarbons, which directly governs oil swelling and mobilization during CO_2_-EOR.^18^ In a second study, a data-driven framework was developed to estimate CO_2_ solubility in brines using brine density as a surrogate for detailed ionic composition. This approach addressed a major limitation in CCUS applications, where complete brine chemistry is often unavailable, and enabled improved prediction of solubility trapping potential during CWF.^19^ In the third study, measurements of CO_2_ solubility were conducted in synthetic brines and produced waters spanning a wide salinity and pressure range.^20^ Collectively, these studies establish a thermodynamic foundation, linking CO_2_ solubility in aqueous and hydrocarbon phases to oil recovery and CO_2_ storage performance. However, while they quantify phase behavior under static conditions, they do not directly capture how solubility-driven mechanisms manifest during multiphase flow in porous media.

Laboratory coreflooding experiments provide a critical bridge between fluid-phase thermodynamics and reservoir-scale performance. Coreflooding studies have demonstrated that CWF can enhance oil recovery relative to conventional WF and promote CO_2_ storage through solubility trapping. Core-scale experiments also allow quantification of pressure drop, recovery factor, and fluid partitioning under reservoir-representative conditions.^21,22^ Nevertheless, coreflooding provides limited direct insight into the pore-scale displacement mechanisms responsible for the observed macroscopic trends.

Pore-scale experimental techniques, particularly microfluidic and micromodel studies, complement coreflooding by enabling direct visualization of multiphase flow, mass transfer, and capillary trapping.^23,24^ Microfluidic experiments have revealed the importance of diffusion-driven CO_2_ transport, transient IFT reduction, and ganglion dynamics in controlling residual oil saturation.^24^ Recent advances have extended pore-scale studies to elevated pressures, allowing investigation of near-miscible CO_2_–oil interactions under reservoir-relevant conditions that are inaccessible using conventional visualization techniques.^23,25^

Despite significant progress, integrated studies that explicitly connect CO_2_ solubility behavior with pore-scale displacement mechanisms and core-scale oil recovery and CO_2_ retention during CWF remain limited. This gap motivates the present work, which integrates the knowledge of CO_2_ solubility in aqueous and hydrocarbon phases with coreflooding and microfluidic experiments to evaluate the potential of CWF for simultaneous EOR and CO_2_ storage in sandstone reservoirs. By linking thermodynamic solubility trends to pore-scale processes and core-scale performance metrics, this study provides mechanistic insight into the design and optimization of carbonated water injection strategies for CCUS applications.

## 2 Materials and Procedure

### 2.1 Materials

The model hydrocarbons used in this study were *n*-hexane with a purity of 99%, supplied by Beantown Chemical, and *n*-decane with a purity of 99%, obtained from Sigma-Aldrich. Crude oil and the corresponding produced water samples were collected from Havvco oil wells located in Oklahoma, United States. Analytical-grade sodium chloride (NaCl) with a minimum purity of 99.0%, purchased from Fisher Scientific, was used to prepare brines of different salinities. Activated alumina powder with a purity of 99.9%, also procured from Fisher Scientific, was employed for the purification of *n*-decane prior to IFT measurements to eliminate surface-active impurities.

For microfluidics-based EOR experiments, Sudan Red 7B (dye content 95%) was procured from Alfa Aesar to dye the oils. Physical rock network chips (porous area dimension: 2 cm × 1 cm) were procured from Micronit Microtechnologies (Enschede, Netherlands). For coreflooding-based EOR experiments, two sandstone reservoir cores, i.e., Berea and Bentheimer (diameter: 25 mm; length: 50 mm) were procured from Kocurek Industries, Inc. Berea sandstone (Product ID: SS-103; Formation: Upper Devonian) typically exhibits a porosity of 18 – 21% and a permeability in the range of 80–120 mD, while Bentheimer sandstone (Product ID: SS-102; Formation: Valanginian) generally shows higher porosity (23–26%) and significantly higher permeability (1,500–3,500 mD). The cores were supplied in standard dimensions, oven-dried, and vacuum-saturated with brine prior to experimental use.

Brine solutions were prepared by dissolving the required amount of NaCl in degassed DI water. The deionized water, having a resistivity of 18.1 MΩ·cm, was degassed using a thermal degassing procedure. The degassing process involved boiling the water to remove dissolved gases, followed by cooling it to ambient temperature in a sealed container to prevent reabsorption of atmospheric gases.^26,27^

### 2.2 Densities of Brine and Produced Water Samples

To determine the porosity of the core samples, accurate measurement of liquid density, and weights of dry and liquid-saturated rock sample and its length and diameter are required. The densities of the prepared brines and produced water samples were measured at 18 °C and atmospheric pressure using a precision densitometer (make: Mettler Toledo; model: DA-100 M).

### 2.3 IFT Measurements

Pendant drop method was employed to measure the IFT of CO_2_-saturated brine–hydrocarbon systems. The experimental setup is illustrated in Figure 1. The apparatus comprised two saturation cells (make: Swagelok; Part #: 316L-HDF4-150; capacity: 150 mL; max. pressure: 1,800 psi) used to saturate brine and oil with CO_2_, respectively. Both cells were connected to a single syringe pump (make: Teledyne ISCO; model: 260D; max. pressure: 10,000 psi) through a tee-junction, ensuring equal pressure exposure in both cells. Utilizing a single pump, rather than two independent pumps, minimizes the risk of pressure discrepancies arising from pump sensitivity variations. The view cell (make: Core Laboratories) was equipped with transparent windows at both ends, allowing direct visualization of the droplet. A diffuse light source was positioned on one side of the cell to provide uniform backlighting, while a CCD camera (make: Ramé-Hart) on the opposite side captured high-contrast images of the pendant drop. Homogeneous illumination was employed to enhance edge detection, thereby improving measurement precision. The *n*-decane supplied by Sigma-Aldrich with a stated purity of 99% was further purified to eliminate trace oxidized species and other surface-active impurities. The purification was carried out by repeatedly passing the *n*-decane through activated alumina until the measured interfacial tension between *n*-decane and water reached a stable equilibrium value. It was found that consistent and reproducible readings were obtained after four successive purification cycles, as reported by Sayed et al. (2019).^28^

**Figure 1:**
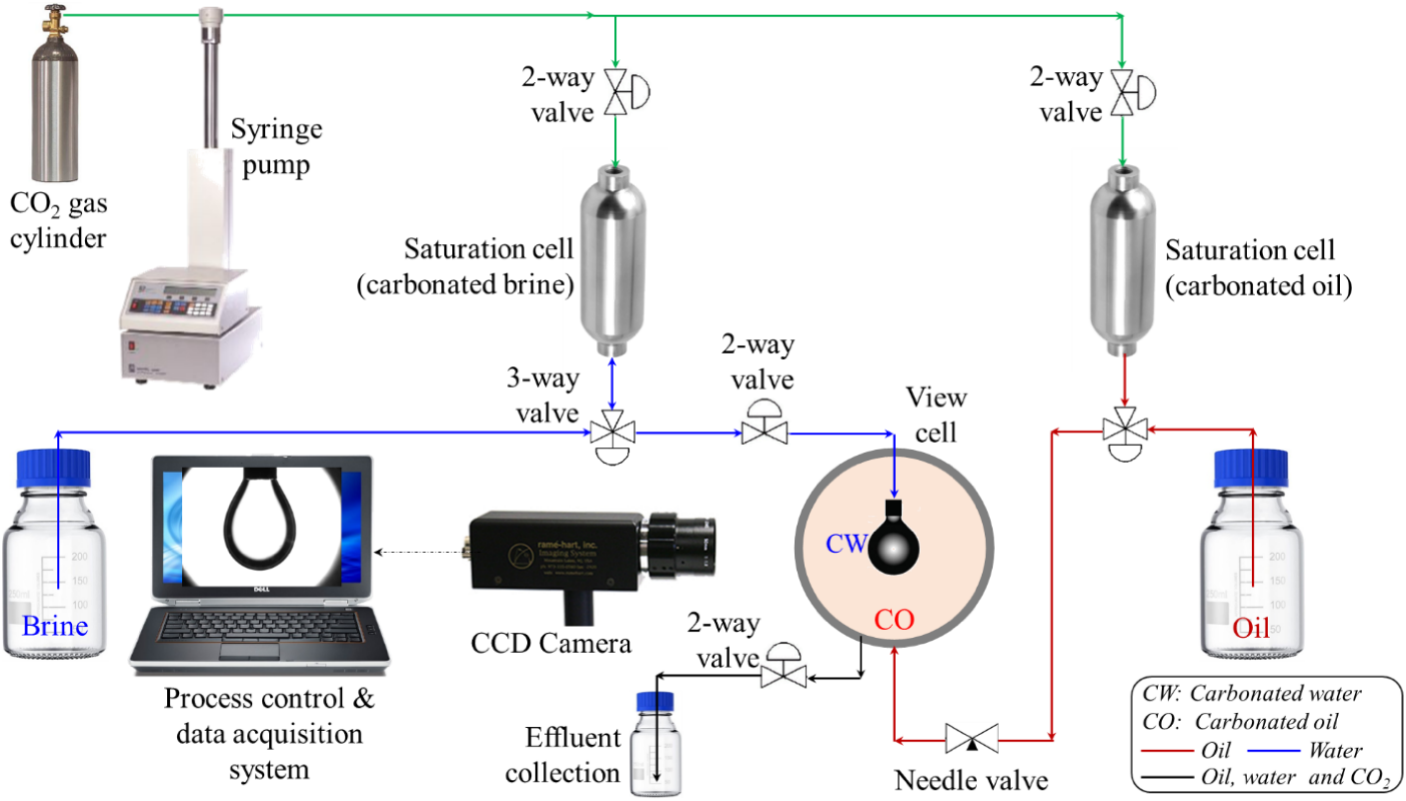
Schematic of the experimental facility for IFT measurements of CO_2_-saturated brine–oil systems.

Equilibrium IFT measurements were performed for CO_2_-saturated brine and CO_2_-saturated *n*-decane at 0, 150, 500, and 700 psig, and 65 °F (18 °C). The brine and oil samples were each saturated with CO_2_ in their respective saturation cells for a minimum of 18 h at the designated pressure. During IFT measurement, the droplet phase consisted of CO_2_-saturated brine, while the surrounding phase was CO_2_-saturated oil. The oil was introduced from the bottom of the view cell, and the brine was injected from the top, given its higher density relative to oil. Each IFT measurement was carried out until it reached a constant value and the data were recorded at 1 min intervals using DROPimage Advanced (make: Ramé-Hart) Software. For each pressure and brine salinity condition, a minimum of three droplets were analyzed and the average values and the corresponding standard deviation IFT values were reported.

### 2.4 Experimental Facility to Conduct Microfluidics-Based EOR Experiments

Figure 2a shows a schematic diagram of the experimental facility used to conduct microfluidics-based EOR experiments with model oil-carbonated model brine, and crude oil-carbonated produced water systems. For each experiment, a microfluidic chip (physical rock network), as shown in Figure 2b, was first completely saturated with oil to achieve 100% oil saturation. A constant back-pressure (test pressures: 150 and 700 psig) was maintained throughout the experiment by using a zero flow back-pressure regulator (BPR) (make: Equilibar, PRT# US20140203198; port size: 1/16 in.; max. pressure: 2,000 psi). Then, the flow direction was changed to the bypass line, and the 6-way selector valve was adjusted to inlet position #2 for brine/water flooding. The brine was then injected through the bypass line until all trapped air was displaced from the line. After that, the flow was redirected to the mainline and the brine injection was continued until the irreducible oil saturation (*S*_oi_) was attained in the chip. Using the same protocol, the chip was then flooded with carbonated water until no more oil was produced and was left overnight for aging. Then the CWF continued to study if any additional oil could be recovered after aging. The oil phase was dyed with Sudan Red 7B at a concentration of 1 g/L, to enhance visual contrast among the phases for fluid saturation measurements. The oil and water injection rates were maintained at 1 μL/min using syringe pumps. The residual oil saturations after each flooding step were analyzed using ImageJ software.

**Figure 2:**
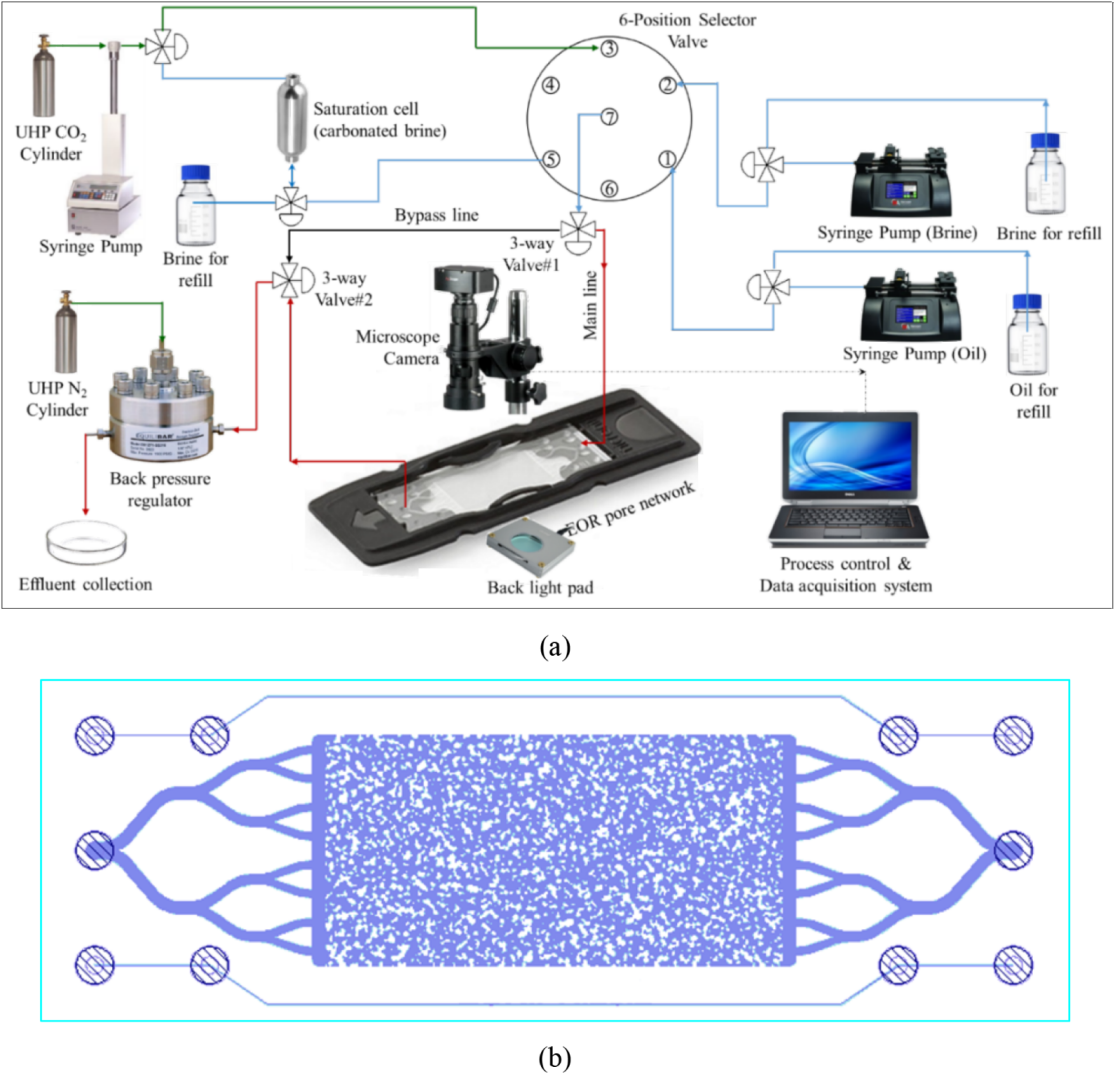
(a) Schematic of the microfluidics-based EOR experimental setup; (b) schematic design of physical rock network chip.

### 2.5 Experimental Facility to Conduct Coreflooding-Based EOR Experiments

Figure 3 shows a schematic diagram of the experimental facility we built to conduct coreflooding-based EOR experiments with model oil–carbonated brine and crude oil–carbonated produced water systems. The coreflooding-based EOR experiments were conducted using two different types of sandstone (Berea and Bentheimer) core samples at pressures of 150 and 700 psig. Before starting each coreflooding experiment, the core sample was saturated with test brine using a vacuum chamber (make: BACOENG) at 28 inHg until constant mass was reached. The porosity was subsequently determined from the mass difference between the dry and saturated states.

**Figure 3:**
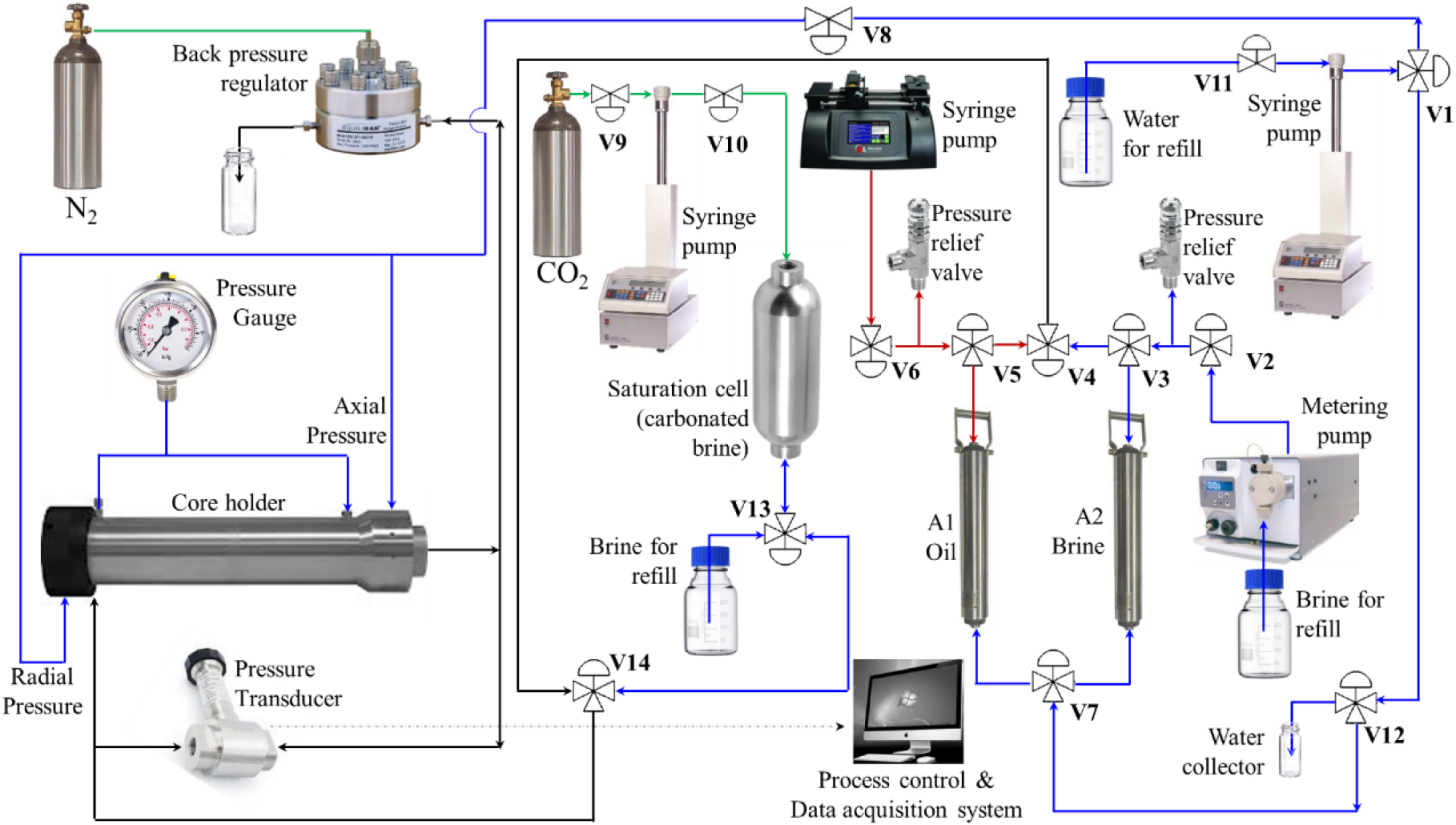
Schematic of the experimental facility to conduct coreflooding experiments for oil–brine/carbonated brine–rock systems.

The core sample saturated with the aqueous phase was installed in the core holder by wrapping using a Teflon tape. 300 psig and 1000 psig confining pressures were maintained for the coreflooding experiments conducted at 150 and 700 psig pressures, respectively. The test brine was injected from accumulator #A2 at a constant flow rate of 1 mL/min to reestablish saturation within the core sample and fill the flow lines. Injection was maintained until no air bubbles were detected at the BPR. Then the system was pressurized to test pressure by engaging the BPR with nitrogen gas. The carbonated brine was prepared in the saturation cell at the test pressure for a minimum of 24 h. The oil accumulator #A1 was pressurized to test pressure using the syringe pump. Then the oil flooding was conducted at 0.1 mL/min until irreducible aqueous phase saturation was achieved. The core was then left overnight for aging. Subsequently, WF was performed at 0.1 mL/min until irreducible saturation was achieved to evaluate oil recovery after WF. This was followed by CWF to investigate the potential for additional oil recovery. The flow rates of both the aqueous and oil phases were maintained at 0.1 mL/min using syringe pumps. A constant back-pressure was ensured throughout the experiment by employing a zero-flow BPR set to the test pressure. Both microfluidics- and coreflooding-based EOR experiments were conducted at 18 °C.

A single EOR experiment was conducted for each condition. This approach was adopted due to the longer duration of each experiment, approximately 3 to 4 days per test, including vacuum saturation of the core samples, a minimum of 24 h for carbonated brine equilibration, oil flooding to irreducible aqueous phase saturation, overnight aging, and sequential WF and CWF stages, and the scope of the test matrix, which comprised more than 30 unique combinations of pressure, brine salinity, oil type, and rock type across the microfluidic and coreflooding experiments.

## 3 Results and Discussion

### 3.1 Density Measurements of Aqueous Phases

To calculate the porosity of the core samples, the densities of DI water, NaCl brines and produced water at 18 °C, were measured using the densitometer, and the density data are shown in Table 1.

**Table 1:** Measured Densities of DI Water, Produced Water and NaCl Brines at Atmospheric Pressure and 18 °C

Sample	Measured density (kg/m^3^)
DI water	998.67
PW1	1013.20
1 M	1034.40
3 M	1106.00
5 M	1166.20

### 3.2 IFT Measurements of CO_2_-Saturated Brine and CO_2_-Saturated Hydrocarbon Systems

The IFT of the *n*-decane–water system was measured to be 51.59 ± 0.28 mN/m at 18 °C and atmospheric pressure, which is in close agreement with previously reported values in the literature. For example, Zhang et al. (2022) reported an IFT of 51.9 mN/m at 25 °C^29^ and Zeppieri et al. (2001) reported values of 52.67 mN/m at 15 °C and 52.33 mN/m at 25 °C.^30^ The close correspondence confirms the accuracy, reproducibility, and reliability of the present experimental setup.

Figures 4a–d show the dynamic evolution of IFT between CO_2_-saturated brine and CO_2_-saturated *n*-decane at different pressures and salinities. The IFT initially exhibited a relatively high value and gradually decreased with time until reaching equilibrium. This transient behavior is attributed to mass transfer and redistribution of CO_2_ between the phases, leading to a gradual approach toward thermodynamic equilibrium at the interface.

**Figure 4:**
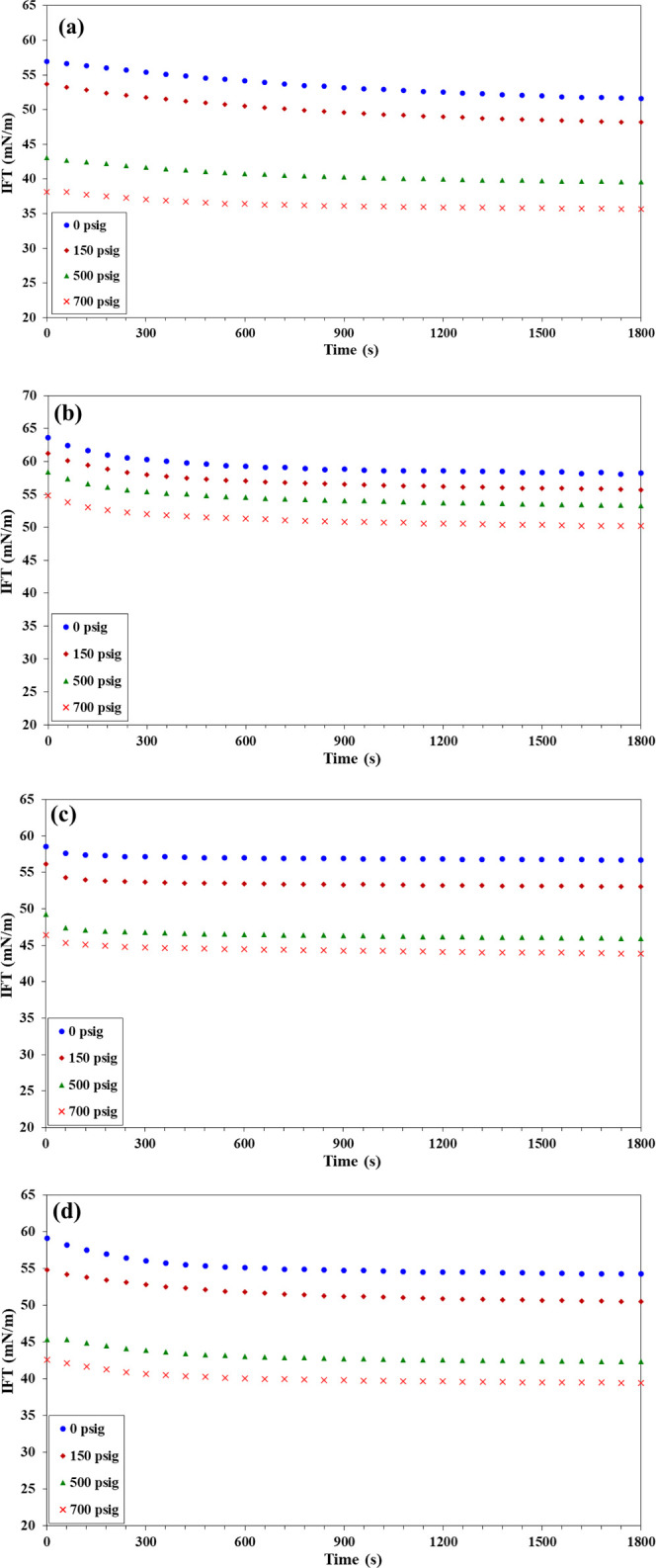
Dynamic IFT of CO_2_-saturated (a) DI water–*n*-decane; (b) 1 M NaCl brine–*n*-decane; (c) 3 M NaCl brine–*n*-decane; (d) 5 M NaCl brine–*n*-decane versus time at various pressures.

The corresponding equilibrium IFT values for all experimental conditions are summarized in Table 2. The variation of equilibrium IFT with pressure is presented in Figure 5a, where a near-linear decrease in IFT is observed as pressure increases from 0 to 700 psig for all salinity levels. This trend reflects enhanced CO_2_ solubility in both the aqueous and hydrocarbon phases at elevated pressures, leading to a reduction in interfacial free energy. As pressure increases, greater CO_2_ dissolution in both brine and oil reduces the compositional contrast between the two phases, thereby lowering the interfacial free energy and leading to a decrease in IFT.

**Table 2:** Summary of Equilibrium IFT Data of CO_2_-Saturated Brine and CO_2_-Saturated *n*-Decane Systems at Various Pressures

	IFT (mN/m)			
Pressure (psig)	DI water	1 M Brine	3 M Brine	5 M Brine
0	51.59 ± 0.28	54.27 ± 0.09	56.72 ± 0.06	58.23 ± 0.20
150	48.19 ± 0.24	50.46 ± 0.26	53.04 ± 0.02	55.71 ± 0.03
500	39.58 ± 0.15	42.32 ± 0.08	45.96 ± 0.16	53.31 ± 0.09
700	35.67 ± 0.12	39.43 ± 0.12	43.84 ± 0.03	50.20 ± 0.04

**Figure 5:**
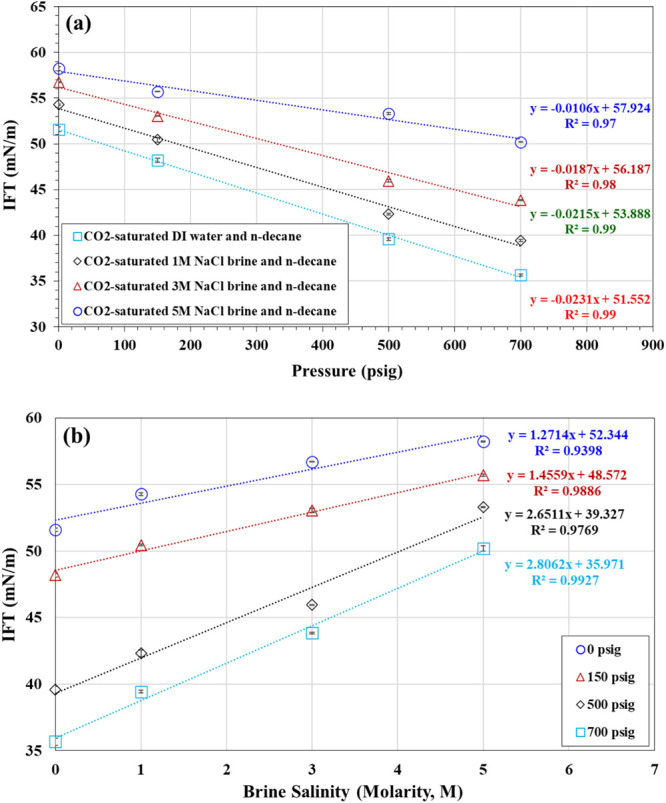
Equilibrium IFT of CO_2_-saturated brines and CO_2_-saturated *n*-decane (a) as a function of pressure; and (b) as a function of NaCl brine salinity.

The dependence of equilibrium IFT on brine salinity is shown in Figure 5b. The results indicate that, at a fixed pressure, the reduction in IFT becomes less pronounced as salinity increases. The phenomenon is explained by the migration of surface-active species from the oil phase to the oil–water interface. The electrical double-layer (EDL) theory provides a more comprehensive explanation: as salinity decreases, the Debye length increases, strengthening the interfacial electric field and promoting the adsorption of polar oil molecules at the interface, which in turn lowers the IFT.^31^ In contrast, high salinity conditions compress EDL, reduce interfacial adsorption of polar species, and consequently yield higher equilibrium IFT values.

### 3.3 Residual Oil Saturations in Microfluidics-Based EOR Experiments

Tables 3–5 summarize residual oil saturations (*S*_or_) of the microfluidics-based EOR experiments conducted with *n*-decane, *n*-hexane, and crude oil, respectively, with carbonated aqueous phases (deionized water, 1 M NaCl brine, 5 M NaCl brine, and produced water), at 150 psig and 700 psig. The oil swelling factors reported in these tables represent the volumetric expansion ratio of oil upon CO_2_ dissolution under pressurized conditions, and therefore directly reflect the extent of CO_2_ solubility in the oil phase. The swelling factor values for each carbonated oil–water system were adopted from our previous work.^18^ In Tables 3–5, the apparent oil saturation obtained from image analysis represents the areal fraction of the pore space occupied by oil during displacement in the microfluidic chip. This value reflects the visually observed oil distribution; however, it includes the effect of CO_2_-induced oil swelling during CWF. To account for this effect, the *S*_or_ was corrected by dividing the apparent oil saturation by the measured swelling factor. The corrected *S*_or_ provides a more representative estimate of the true remaining oil volume after CWF.

**Table 3:** *S*_or_ of *n*-Decane in Microfluidics-Based EOR Experiments Post-WF and CWF with Different Aqueous Phases (DI Water, 1 M Brine, 5 M Brine)^a^

	DI water at 150 psig		DI water at 700 psig	
Flooding	Apparent oil saturation	Actual oil saturation *Swelling factor: 1.03*	Apparent oil saturation	Actual oil saturation *Swelling factor: 2.2*
Oil	1.00	1.00	1.00	1.00
Water	0.26	**0.26**	0.32	**0.32**
CW1	0.29	**0.28**	0.31	**0.14**
CW2	0.29	0.29	0.32	0.14
CO_2_	0.17	0.17	0.08	0.04

	5 M Brine at 150 psig		5 M Brine at 700 psig	
Flooding	Apparent oil saturation	Actual oil saturation *Swelling factor: 1.03*	Apparent oil saturation	Actual oil saturation *Swelling factor: 2.05*
Oil	1.00	1.00	1.00	1.00
5 M Brine	0.15	**0.15**	0.21	**0.21**
5 M CB1	0.14	**0.14**	0.23	**0.11**
5 M CB2	0.14	0.13	0.24	0.11
CO_2_	0.11	0.10	0.07	0.03

	1 M Brine at 150 psig		1 M Brine at 700 psig	
Flooding	Apparent oil saturation	Actual oil saturation *Swelling factor: 1.03*	Apparent oil saturation	Actual oil saturation *Swelling factor: 2.15*
Oil	1.00	1.00	1.00	1.00
1 M Brine	0.26	**0.26**	0.26	**0.26**
1 M CB1	0.28	**0.27**	0.34	**0.16**
1 M CB2	0.27	0.26	0.30	0.14
CO_2_	0.16	0.15	0.08	0.04

a CW1/CB1: carbonated water or brine flooding, before aging; CW2/CB2: carbonated water or brine flooding, after aging.

**Table 4:** *S*_or_ of *n*-Hexane in Microfluidics-Based EOR Experiments Post-WF and CWF with Different Aqueous Phases (DI Water, 1 M Brine, 5 M Brine)

	DI water at 150 psig		DI water at 700 psig	
Flooding	Apparent oil saturation	Actual oil saturation *Swelling factor: 1.05*	Apparent oil saturation	Actual oil saturation *Swelling factor: 5.73*
Oil	1.00	1.00	1.00	1.00
Water	0.42	**0.42**	0.31	**0.31**
CW1	0.24	**0.23**	0.31	**0.05**
CW2	0.24	0.23	0.29	0.05
CO_2_	0.01	0.01	0.01	0.00

	5 M Brine at 150 psig		5 M Brine at 700 psig	
Flooding	Apparent oil saturation	Actual oil saturation *Swelling factor: 1.06*	Apparent oil saturation	Actual oil saturation *Swelling factor: 4.84*
Oil	1.00	1.00	1.00	1.00
5 M Brine	0.32	**0.32**	0.17	**0.17**
5 M CB1	0.25	**0.24**	0.16	**0.03**
5 M CB2	0.26	0.24	0.16	0.03
CO_2_	0.09	0.09	0.02	0.00

	1 M Brine at 150 psig		1 M Brine at 700 psig	
Flooding	Apparent oil saturation	Actual oil saturation *Swelling factor: 1.05*	Apparent oil saturation	Actual oil saturation *Swelling factor: 5.5*
Oil	1.00	1.00	1.00	1.00
1 M Brine	0.26	**0.26**	0.30	**0.30**
1 M CB1	0.18	**0.17**	0.24	**0.04**
1 M CB2	0.18	0.17	0.21	0.04
CO_2_	0.02	0.01	0.01	0.00

CW1/CB1: carbonated water or brine flooding, before aging; CW2/CB2: carbonated water or brine flooding, after aging.

**Table 5:** *S*_or_ of Havvco 1 Crude Oil in Microfluidics-Based EOR Experiments Post-WF and CWF **^a^**

	Produced water at 150 psig		Produced water at 700 psig	
Flooding	Apparent oil saturation	Actual oil saturation *Swelling factor: 1.01*	Apparent oil saturation	Actual oil saturation *Swelling factor: 1.31*
Crude oil	1.00	1.00	1.00	1.00
Produced water	0.23	**0.23**	0.21	**0.21**
CW1	0.23	**0.22**	0.09	**0.07**
CW2	0.26	0.26	0.09	0.07
CO_2_	0.05	0.05	0.04	0.03

a CW1: carbonated produced water flooding, before aging; CW2: carbonated produced water flooding, after aging.

When *n*-decane was used as the oil phase, CWF at 150 psig resulted in negligible change in *S*_or_, indicating limited CO_2_ dissolution and insufficient oil swelling at low pressure. However, at 700 psig, *S*_or_ decreased by approximately 10–18% following CWF, which is attributed to significantly enhanced CO_2_ solubility in *n*-decane, leading to increased oil swelling, viscosity reduction, and mobilization of residual oil confined by capillary forces.

When *n*-hexane was used as the oil phase, substantial reductions in *S*_or_ were observed at both pressures (Table 4), with decreases of 9–19% at 150 psig and 14–26% at 700 psig post-CWF. The markedly larger swelling factors of *n*-hexane, particularly at 700 psig, indicate strong CO_2_–oil affinity, resulting in higher oil swelling. This behavior highlights the dominant role of oil volatility and CO_2_ solubility in governing displacement efficiency in lighter hydrocarbon systems.

When Havvco 1 crude oil was used as the oil phase, CWF reduced residual oil saturation (*S*_or_) by approximately 4% at 150 psig and 25% at 700 psig, as can be seen from Table 5. The comparatively smaller swelling factors observed for crude oil reflect its complex multicomponent composition and limited CO_2_ solubility relative to *n*-alkanes, particularly at lower pressure. However, at 700 psig, additional CO_2_ dissolution along with reduced IFT and improved oil mobility led to a decrease in *S*_or_.

Across all systems, *S*_or_ following direct CO_2_ flooding decreased irrespective of experimental pressure, indicating that free-phase CO_2_ injection provides additional displacement mechanisms, including oil swelling, viscosity reduction, and possible local miscibility effects, which are not fully realized during CWF alone. Figure 6 shows that oil swelling becomes more pronounced as pressure increases from 150 to 700 psig, as evidenced by the expansion of the red-colored regions corresponding to the oil phase (*n*-decane) identified through ImageJ analysis.

**Figure 6:**
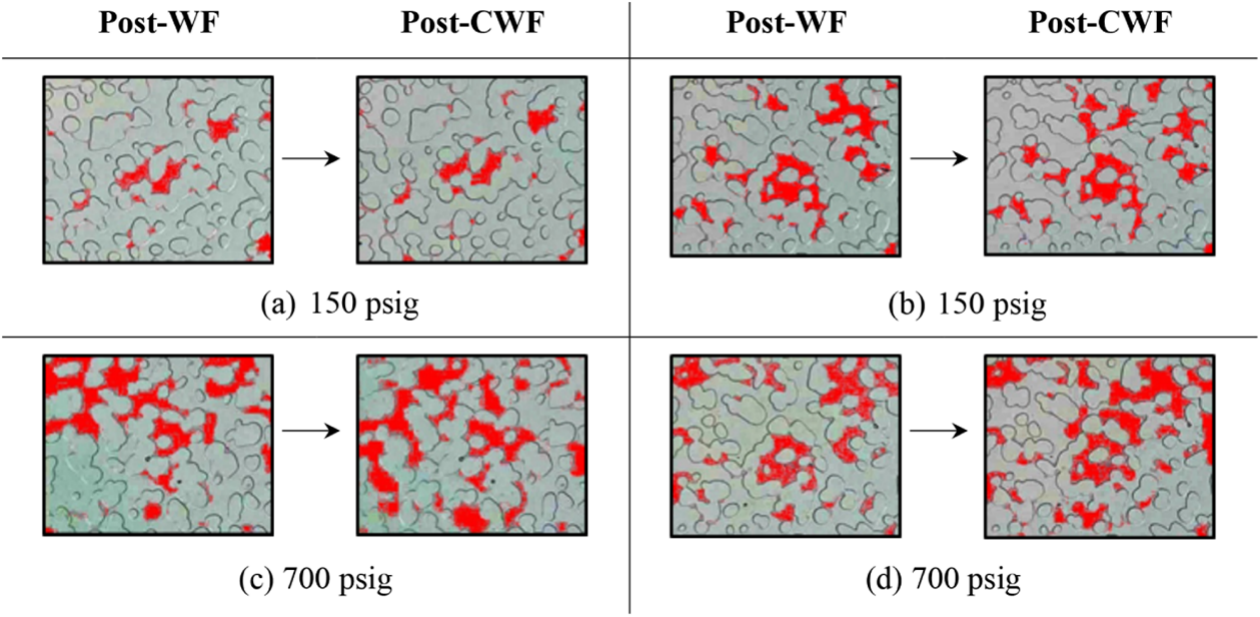
Evidence of oil (*n*-decane) swelling post-CWF (the red colored areas indicate oil saturation processed using ImageJ) at two different locations in the microfluidic chip. (a) and (b) correspond to two different locations in the microfluidic chip at 150 psig, both showing a modest increase in oil swelling and redistribution post-CWF compared to post-WF conditions. (c) and (d) correspond to two different locations at 700 psig, where a more significant increase in oil swelling observed post-CWF, indicating enhanced swelling at higher pressure.

This increased oil volume is attributed to enhanced CO_2_ solubility at higher pressure, which promotes oil swelling and contributes to the mobilization of capillary-trapped oil. In contrast, at 150 psig, limited CO_2_ dissolution results in minimal observable swelling. Figures 7a–b present representative microfluidic images at 700 psig post-CWF followed by post-CO_2_ flooding, respectively, and the corresponding *S*_or_ data are provided in Table 3.

**Figure 7:**
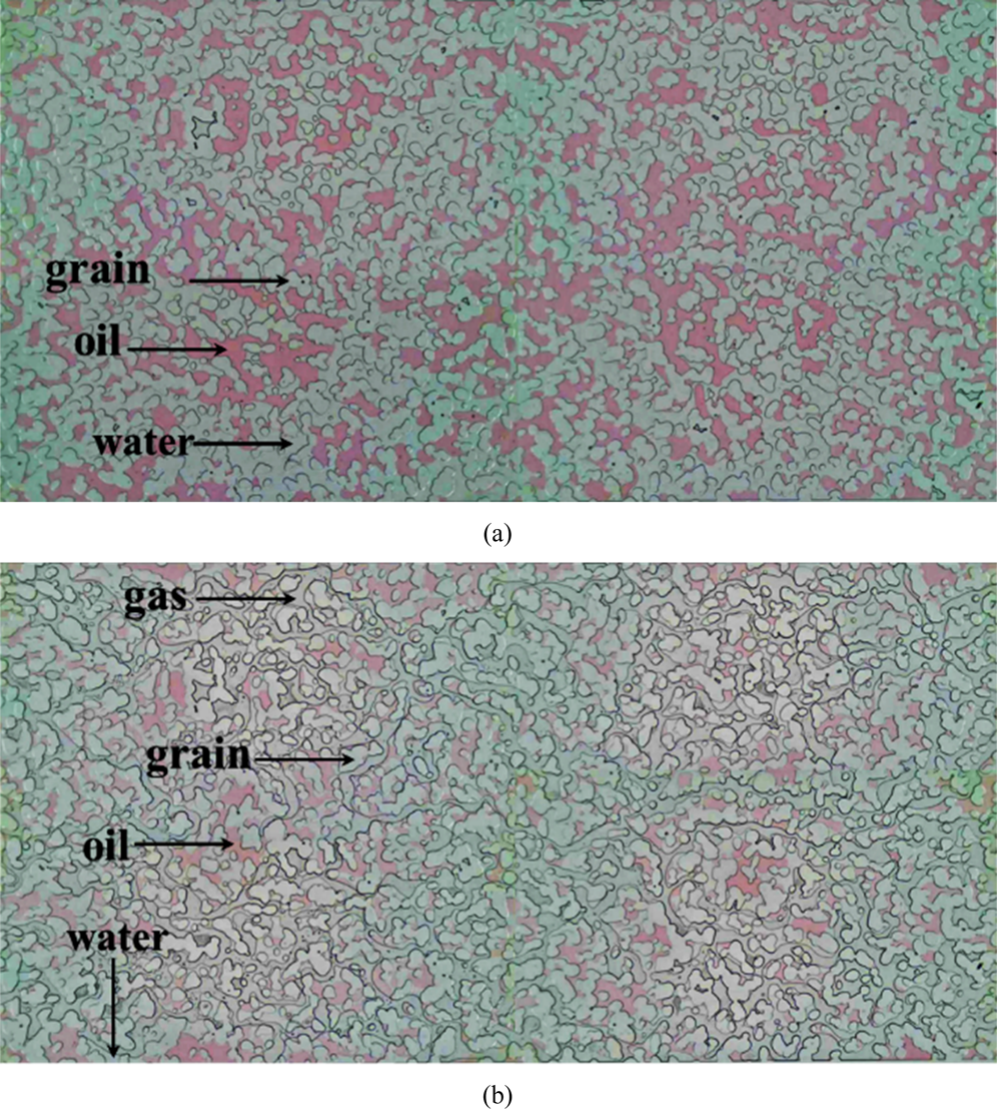
Chip-scale images (a) post-CWF at 700 psig; and (b) post-CO_2_ flooding at 700 psig.

### 3.4 Residual Oil Saturations in Coreflooding-Based EOR Experiments

Table 6 summarizes the oil recovery (expressed as a percentage of the original oil in place, OOIP) from coreflooding experiments conducted at 150 and 700 psig. In addition, the number of moles of CO_2_ sequestered in the oil and water phases within the Berea and Bentheimer core samples are also presented in the table. The effects of brine salinity, injection pressure, and core type on oil recovery are illustrated in Figure 8.

**Table 6:** Summary of Oil Saturations Post-WF and Post-CWF (% OOIP), and Number of Moles of CO_2_ Captured in Oil and Water Phases Present inside the Core samples^a^

Type of core	Aqueous-oil phases	*S*_or**‑**wf_	*S*_or**‑**cwf_	*S*_or:wf**+**cwf_	*n*_CO2**‑**brine_	*n*_CO2**‑**oil_	*n*_CO2**‑**oil**+**CO2**‑**brine_
150 psig							
Berea	Water–*n*-decane	41.83	8.31	50.14	0.00060	0.00008	0.00068
	1 M brine–*n*-decane	33.53	6.04	39.58	0.00045	0.00009	0.00054
	5 M brine–*n*-decane	25.21	2.77	27.98	0.00021	0.00012	0.00033
	PW1–CO	43.93	12.46	56.39	0.00058	0.00007	0.00065
700 psig							
Berea	Water–*n*-decane	54.75	13.57	68.33	0.00205	0.00203	0.00408
	1 M brine–*n*-decane	48.34	11.07	59.41	0.00132	0.00319	0.00451
	5 M brine–*n*-decane	34.87	3.83	38.70	0.00045	0.00464	0.00509
	PW1–CO	45.37	19.00	64.37	0.00262	0.00015	0.00277
150 psig							
Bentheimer	Water–*n*-decane	48.72	14.25	62.96	0.00084	0.00006	0.00090
	1 M brine–*n*-decane	44.04	12.17	56.20	0.00062	0.00008	0.00071
	5 M brine–*n*-decane	31.97	6.80	38.78	0.00030	0.00013	0.00043
	PW1–CO	44.57	13.30	57.87	0.00054	0.00005	0.00059
700 psig							
Bentheimer	Water–*n*-decane	67.58	17.46	85.04	0.00321	0.00174	0.00495
	1 M brine–*n*-decane	57.24	14.25	71.50	0.00163	0.00348	0.00511
	5 M brine–*n*-decane	39.58	9.06	48.64	0.00060	0.00493	0.00553
	PW1–CO	54.68	18.13	72.81	0.00167	0.00026	0.00193

a **Notations**: *S*_or‑wf_: residual oil-saturation post-WF; *S*_or‑cwf_: residual oil-saturation post-CWF; *S*_or,wf+cwf_: residual oil-saturations post-WF and CWF (*S*_or:wf+cwf_ = *S*_or‑wf_+ *S*_or‑cwf_); *n*_CO2‑brine_: no. of moles of CO_2_ in brine, post-CWF; *n*_CO2‑oil_: no. of moles of CO_2_ in oil, post-CWF; *n*_CO2‑oil+CO2‑brine_: total number of moles of CO_2_ present in the core sample post-CWF; PW1: produced water obtained from Havvco 1 oil well, CO: crude oil from Havvco 1 oil well.

**Figure 8:**
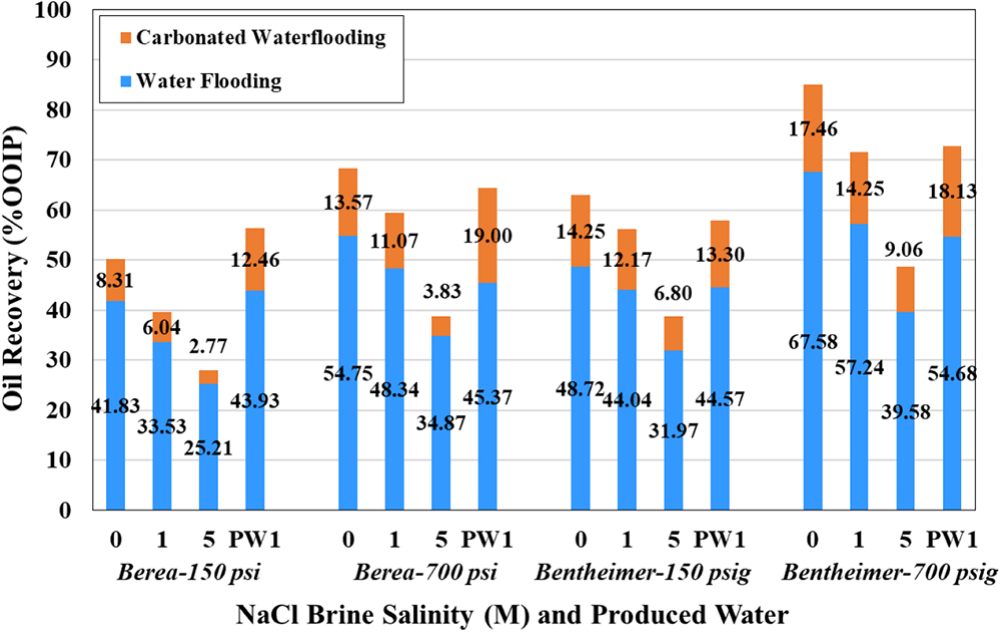
Effect of NaCl brine salinity on oil recovery at 150 and 700 psig with Berea and Bentheimer cores.

The results indicate that low-salinity waterflooding (LSWF) yields a higher incremental oil recovery than high-salinity waterflooding (HSWF). This improvement is attributed to salinity-induced wettability alteration toward more water-wet conditions, which reduces capillary forces and enhances oil mobility.^32^ Additionally, LSWF can induce controlled fines migration and expand the electrical double layer at the rock–fluid interface, thereby weakening oil–rock adhesion and improving microscopic sweep efficiency.^33^ These physicochemical mechanisms are suppressed under high-salinity conditions due to electrical double-layer compression, resulting in lower oil recovery during HSWF. A similar salinity-dependent trend was observed for oil recovery following CWF, indicating that low salinity continues to enhance displacement efficiency even in the presence of dissolved CO_2_. Higher *S*_or_ was observed at 150 psig compared to 700 psig, which can be attributed to reduced CO_2_ solubility in oil at lower pressures. Lower CO_2_ dissolution limits oil swelling, viscosity reduction, and IFT reduction, which are primary mechanisms responsible for mobilizing trapped oil during CWF. At 700 psig, increased CO_2_ solubility significantly enhances these effects, leading to improved oil mobility, reduced capillary trapping, and consequently higher oil recovery.

Bentheimer sandstone consistently exhibited higher oil recovery than Berea sandstone when pressure, salinity, and oil type were kept constant. This behavior is attributed to the higher permeability and more homogeneous pore structure of Bentheimer sandstone, which facilitates more uniform fluid distribution and efficient displacement during both WF and CWF. In contrast, Berea sandstone exhibits lower permeability and greater mineralogical and pore-scale heterogeneity, which can promote preferential flow paths, early water breakthrough, and higher *S*_or_.

The effect of NaCl brine salinity on total CO_2_ sequestered, in moles per cc of pore volume (PV), at 150 and 700 psig using Berea and Bentheimer cores is shown in Figure 9. From Figure 9, it can be observed that at 700 psig, high-salinity carbonated waterflooding (HSCWF) results in lower oil recovery than low-salinity carbonated waterflooding (LSCWF), leading to higher *S*_or_ within the core samples. At elevated pressure, CO_2_ exhibits substantially greater solubility in oil than in brine, and therefore the increased *S*_or_ under HSCWF conditions provides a larger dissolution sink for CO_2_. As a result, the increased oil saturation facilitated enhanced CO_2_ retention within the core, leading to greater overall CO_2_ sequestration in the high-salinity flooding scenario despite poorer oil recovery.

**Figure 9:**
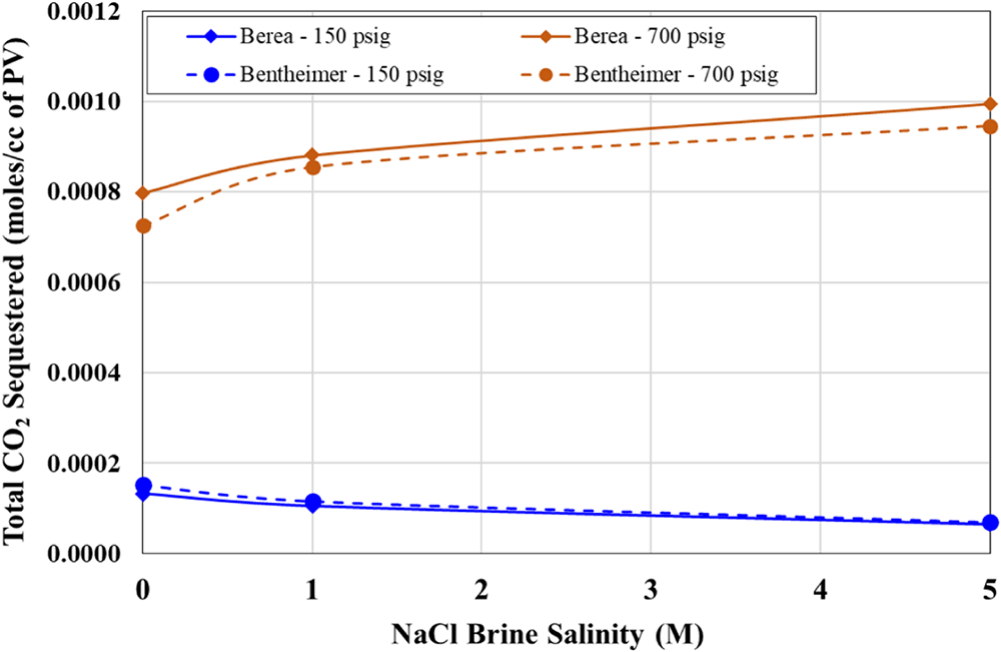
Effect of NaCl brine salinity on total CO_2_ sequestered (moles per cc of PV) at 150 and 700 psig with Berea and Bentheimer sandstone core samples.

Conversely, at 150 psig, the absolute solubility of CO_2_ in both oil and brine is significantly reduced, limiting the contribution of solubility trapping. Under these conditions, LSCWF more effectively enhances oil recovery by promoting wettability alteration toward water-wet conditions and improving microscopic displacement efficiency, which reduces *S*_or_ and alters pore-scale fluid distribution. Although HSCWF retains more residual oil at 150 psig, the reduced CO_2_ solubility and weaker CO_2_–oil interactions at this pressure limit CO_2_ dissolution and retention within the oil phase. Consequently, CO_2_ sequestration is higher after LSCWF than HSCWF at 150 psig, indicating that pore-scale trapping and improved sweep efficiency dominate over solubility trapping at lower pressure.

During carbonated produced water flooding experiments using the corresponding crude oil, substantial incremental oil recovery was observed (Table 6). One of the primary contributing factors is attributed to the significantly lower IFT between produced water and crude oil, measured at approximately 2 mN/m. The lower IFT is indicative of surface-active species present in the produced water, likely originating from residual production chemicals and/or naturally occurring surfactants. When combined with CO_2_ dissolution effects, these surfactants act synergistically to improve oil recovery and influence CO_2_ retention through altered fluid distribution and enhanced trapping within the pore space.

## 4 Conclusion

This study provides a comprehensive experimental evaluation of CWF as a strategy for EOR and geological CO_2_ storage in sandstone reservoirs. By integrating IFT measurements, microfluidic visualization, and coreflooding experiments under controlled pressure and salinity conditions, this work establishes a mechanistic link between pore-scale displacement behavior and core-scale oil recovery and CO_2_ storage.

IFT measurements demonstrated that CO_2_–saturated brine–oil systems exhibit a near-linear reduction in IFT with increasing pressure, while increasing brine salinity counteracts this reduction due to electrical double-layer compression and suppressed interfacial adsorption. Microfluidic experiments revealed that CWF was ineffective for EOR at low pressure (150 psig) due to limited CO_2_ dissolution, whereas at elevated pressure (700 psig), enhanced CO_2_ solubility promotes oil swelling, viscosity reduction, and significant reductions in *S*_or_ (12–25%), depending on the type of hydrocarbons. Lighter hydrocarbon (*n*-hexane) exhibited stronger responses due to higher CO_2_ solubility, while heavier hydrocarbons (*n*-decane and crude oil) required elevated pressure to realize comparable benefits.

Coreflooding experiments confirmed these trends at the continuum scale. LSCWF consistently outperformed HSCWF in terms of oil recovery. At high pressure, HSCWF, despite poorer oil recovery, resulted in greater total CO_2_ sequestration due to increased *S*_or_, which acted as a dominant sink for CO_2_ dissolution. In contrast, at low pressure, limited CO_2_ solubility reduced the effectiveness of solubility trapping, and LSCWF achieved superior CO_2_ retention by enhancing sweep efficiency and pore-scale trapping.

The comparison between Berea and Bentheimer sandstones further highlighted the role of rock properties, with higher permeability and pore-scale homogeneity in Bentheimer cores enabling greater oil recovery and CO_2_ storage. Carbonated produced water flooding of crude oil yielded substantial incremental recovery, attributed to synergistic effects of dissolved CO_2_ and surface-active species present in produced water and crude oil, underscoring the importance of realistic reservoir fluids in CCUS evaluations.

## Data Availability

Data will be made available on request.
